# Strong Surface Orientation Dependent Thermal Transport in Si Nanowires

**DOI:** 10.1038/srep24903

**Published:** 2016-04-26

**Authors:** Yanguang Zhou, Yuli Chen, Ming Hu

**Affiliations:** 1 Aachen Institute for Advanced Study in Computational Engineering Science (AICES), RWTH Aachen University, 52062 Aachen, Germany; 2 Institute of Solid Mechanics, Beihang University (BUAA), Beijing 100191, China; 3 Institute of Mineral Engineering, Division of Materials Science and Engineering, Faculty of Georesources and Materials Engineering, RWTH Aachen University, Aachen 52064, Germany

## Abstract

Thermoelectrics, which convert waste heat to electricity, offer an attractive pathway for addressing an important niche in the globally growing landscape of energy demand. Research to date has focused on reducing the thermal conductivity relative to the bulk. Si nanowires (NWs) have received exceptional attention due to their low-dimensionality, abundance of availability, and high carrier mobility. From thermal transport point of view, the thermal conductivity of Si NWs strongly depends on the detailed surface structure, such as roughness and surface orientation. Here, direct molecular dynamics simulations and theoretical models are used to investigate the thermal transport in Si NWs with diverse surface orientations. Our results show that the thermal conductivity of Si NWs with different surface orientation can differ by as large as 2.7~4.2 times, which suggests a new route to boost the thermoelectric performance. Using the full spectrum theory, we find that the surface orientation, which alters the distribution of atoms on the surface and determines the degree of phonon coupling between the core and the surface, is the dominant mechanism. Furthermore, using spectral thermal conductivity, the remarkable difference in the thermal conductivity for different surface orientation is found to only stem from the phonons in the medium frequency range, with minor contribution from low and high frequency phonons.

Controlling phonon transport in nanowires is of great importance to develop the new generation of thermoelectric devices^1^,^2^,^3^,^4^,^5^,^6^,^7^. The thermoelectric efficiency of such devices is characterized by the figure-of-merit *ZT* = *S*^*2*^*σT*/*κ*, where *S* is the Seebeck coefficient, *σ* is the electrical conductivity, *κ* is the thermal conductivity, and *T* is the system temperature. For NWs with diameter smaller than 100 nm, heat transport (mainly contributed by phonons) becomes more important since the thermal conductivity of low dimensional nanostructures is found to decrease remarkably with respect to their bulk counterparts, while the power factor (*S*^*2*^*σ*) only change slightly^1^,^2^. This means the NWs can be excellent candidates to manufacture the thermoelectric devices with high *ZT* values^7^,^8^,^9^. Due to such characteristics of NWs, the Si-based thermoelectric devices with high *ZT* values have been obtained via incorporating the NWs or nanomesh structures^10^,^11^. The phonon confinement effects in Si NWs mainly originate from the surface scattering, and therefore, strongly depend on the surface structure and the diameter of the NWs^12^,^13^,^14^,^15^,^16^,^17^,^18^,^19^,^20^. Earlier experiments show that the different surface structures lead to remarkable variation in the thermal conductivity of Si NWs with same diameter^6^,^20^. Recently, Martin *et al.*^19^ and Aksamija *et al.*^21^ proved theoretically the phonon-boundary scattering is related to the root mean square of the surface roughness and the phase difference which is the angle between the incoming phonon and the normal direction of the surface. In their work, they found that the thermal conductivity of NWs would be reduced by several times with only a small difference in the surface structure. Meanwhile, surface morphology controlling (e. g. roughness and orientation) has already been realized in experiments successfully^22^,^23^,^24^,^25^,^26^,^27^,^28^. The surface orientation has been found in the past to profoundly affect the mechanical and electronic transport properties of NWs^29^,^30^,^31^. Frederic^32^ also found that the difference in the thermal conductivity of NWs with various faceting surfaces can be as large as about 2 times. However, to the best of our knowledge, the effect of the surface orientation coupled with the surface roughness on the thermal transport in NWs is still not fully understood yet.

In this paper, using atomistic simulations and theory predictions, we examine the impact of surface orientation on the thermal conductivity of Si NWs along the [001] direction (see schematic in Fig. 1). Effects of length and diameter on the thermal conductivity with various oriented surfaces are systematically investigated. Our study shows that the surface orientation can alter the thermal conductivity of Si NWs by as large as 2.7~4.2 times. Detailed analysis of vibrational density of states (VDOS) and spectral thermal conductivity, which was proposed by us recently, are used to reveal the underlying mechanism responsible for this huge difference. The coupling between the surface atoms and the interior (core) atoms, which is the so-called phonon-boundary scattering, is found to be the governing mechanism of the different thermal conductivity of the NWs with various orientation. In addition, we find that only the phonons in the medium frequency region (1.5~10 THz) are affected by surface strongly.

## Simulation Results

The thermal conductivity of Si NWs with 28 different surface orientations is collected and compared in Fig. 2. It is interesting to find that the difference in the thermal conductivity between diverse oriented surfaces can be as large as about 2.7 times (even 4.2 times for Si NWs with smaller cross-section, details will be shown later). Since the only difference between different models is the surface orientation, then it is intuitive to see that the surface orientation should be responsible for the immense difference shown in Fig. 2. Based on Eq. (9) in the theory part, the relaxation time for a specific phonon boundary scattering can be affected by **n**, *φ* and Δ, which means the direction vector of the boundary, the angle between the phonon wave vector and the direction vector of the boundary surface, and the roughness of the surface, respectively. Since the second parameter *φ* is only related to **n**, then the Eq. (9) is only affected by **n** and Δ. Thus, the orientation of the surface can affect the thermal conductivity of Si NWs through two ways: (1) surface orientation can change the angle between the phonon wave vector and the direction vector of the boundary surface (*φ*), then alter the modal specularity and the time that a phonon needs to travel ballistically before hitting the boundary, and therefore, lead to the change of thermal conductivity; (2) different surface orientation corresponds to different distribution of atoms on the surface, and then changes the roughness of the surface, and thus, varies the thermal conductivity of NWs.

To assess the relative contribution of these two effects on the overall thermal transport in Si NWs, we choose two typical models with (100) and (110) surfaces as examples. Using our theoretical model, we can obtain the thermal conductivity of these two models with different RMS height of roughness (Δ). The results are presented in Fig. 3. We find that the largest difference between the two models which have the same Δ is only about 4 W/mK, which is about 5% of the average thermal conductivity of the two mentioned NWs. On the other hand, we notice that a small difference between the Δ of the two NWs can lead to a big change of thermal conductivity (see point A and B in Fig. 3). Therefore, we conclude that the main reason for the large difference of the thermal conductivity of NWs with different surface orientation (Fig. 2) should be the second effect as we discussed above.

Meanwhile, it is well known that the results of NEMD simulations are strongly length dependent along the heat current direction^33^,^34^. Furthermore, the thermal conductivity of NWs is also affected by the size of cross-section intensively^2^,^4^,^20^. We now discuss how these two effects couple with the surface orientation in sequence.

### Length dependence of thermal conductivity of Si NWs

Generally speaking, there is always length effect in NEMD simulations, since the long mean free path phonons are truncated by the length boundaries and contribute little to the overall thermal conductivity^35^. For NWs with cross-sections of a few nanometers, the mean free path (MFP) is only several nanometers^36^. Furthermore, using the method generated by Schelling *et al.*^33^, we also obtain the MFP to be 13.5 nm (300 K), 10.8 nm (500 K) and 9.4 nm (800 K) for the NW with (110) surface orientation, and to be 7.30 nm (300 K), 8.38 nm (500 K) and 5.56 nm (800 K) for the NW with (100) surface orientation. Thus, the linear extrapolation between the reciprocal of thermal conductivity (1/*κ*) and the reciprocal of length (1/*L*) can be used to obtain the thermal conductivity of infinitely long NWs (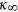) in our simulations, which is the popular method to eliminate the length effect in NEMD simulations^34^,^37^. Here, the system with cross-section of 4.35 ×  4.35 nm^2^ is used as a representative to analyze the length effect of Si NWs. The results are reported in Fig. 4. We find that 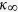 of Si NW with (100) surface orientation is 16.86 W/mK (300 K), 14.39 W/mK (500 K) and 10.25 W/mK (800 K). Wang *et al.*^36^ report the 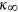 of Si NW with same surface orientation is 6.86 W/mK (400 K). The difference between their results and ours can be attributed to the different potentials used (Stillinger-Weber potential in their work and Tersoff potential in this work), the lattice parameter and the system temperature. Furthermore, in Wang’s^36^ work quantum corrections are used for their results. However, Turney *et al.*^38^ prove that the classical thermal conductivity should not be quantum corrected based on the mode dependence of phonon properties. For NW with (110) surface, *κ*_∞_ is 45.55 W/mK (300 K), 32.26 W/mK (500 K) and 20.83 W/mK (800 K). It can be found that for NWs with infinite length, there is still large difference in thermal conductivity between different surface orientations. In addition, our simulation results are much higher than that of experiments^2^,^4^, since the NWs in our model are perfect (no impurities, defects, and grain boundaries) and the RMS height of roughness is much smoother than that in experiments.

### Cross-section dependence of thermal conductivity of Si NWs

**Figure 1:**
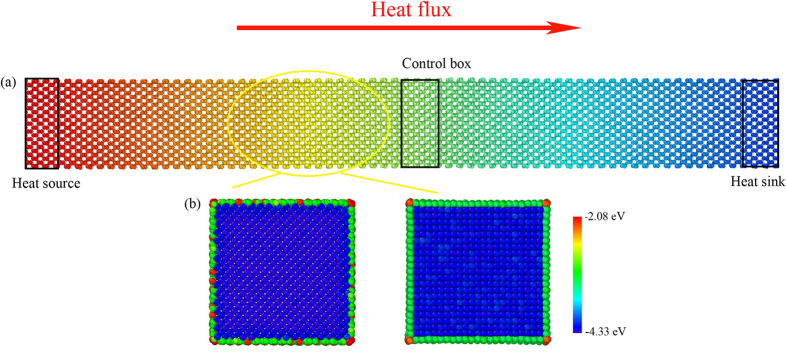
(**a**) Schematic of NEMD simulation domain. The heat source, heat sink, and the control volume used for our FDDDM analysis are highlighted by the rectangular box. The heat flux is along the [001] direction. (**b**) The cross-sectional view of two typical Si nanowires with (100) and (110) surface orientation. The atoms are colored by their atomic potential energy.

It is obvious that for the same orientations/roughness the NW with larger cross-section has higher thermal conductivity, since phonons scatter with boundary less intensively in the case of larger cross-section. All the results of the NWs with different cross-section in this paper are found to be consistent with the common knowledge (Fig. 5). Furthermore, as discussed before, the roughness for the NWs with the same size of cross-section is the key point that leads to the different thermal conductivity. However, it is quite difficult to measure the RMS height of roughness, since the surfaces are already atomically smooth in some cases. Thus, it is necessary to define a parameter which is related to the roughness to describe the roughness dependent thermal conductivity. The surface energy is a quite popular parameter to depict the mechanical properties of NWs with different surface orientation^39^,^40^,^41^,^42^. Unfortunately, we do not find any clear relationship between the thermal conductivity and the surface energy. Since the roughness characterizes the local atom arrangement or structure on the surface region of NWs, we define the average surface atom energy which uses the surface energy divided by the number of atoms in the surface region. Here, we regard the atoms with energy 0.45 eV larger than that of the bulk counterpart as surface region. We find that the thermal conductivity of NWs decreases with the average surface atom energy, since the smaller the average surface atom energy, the more stable the surface structure, which means the boundary scattering decreases in this case. In addition, we can obtain that the thickness of the surface region is about 0.1~0.5 nm, which means the RMS height of roughness should fall in this range as well. From Fig. 1b, it can be also found that the roughness of the NWs in our simulations should be roughly 1~2 atomic layers. Then, using our theoretical model, we can plot the thermal conductivity of NWs vs. RMS height of roughness (Fig. 5). We find that the theoretical predictions are in good agreement with the MD results. Both our theoretical predictions and MD results show that the difference in the thermal conductivity between different surface orientations can be as large as about 3 folders.

It should be noted that the phonon dispersion will change when the cross-section of nanowires (NWs) is quite small^43^,^44^, which is also well known due to the phonon confinement effect. However, this phonon confinement effect is not easy to consider in the theoretical model due to the large computational cost. We calculated the phonon dispersion of Si NWs with cross-section of 4 × 4 u.c. and 6 × 6 u.c. for (100) surface orientation (results not shown for brevity). We find that the small diameter NWs have very crowded and complex phonon band structures. In addition, it is well known that there are two methods to calculate the thermal conductivity of NWs with no impurities in the theoretical processes: 1) Using the bulk phonon dispersions, the total phonon scattering process includes phonon-phonon scattering, phonon-boundary scattering *et al.*, and then the phonon relaxation time can be obtained using the Matthiessen’s rule^21^,^45^,^46^,^47^. 2) Using the full phonon dispersions of NWs, the total phonon relaxation time can be obtained by only considering phonon-phonon scattering in the materials^48^,^49^. Here, we choose the first method to deal with the problem in our cases, because we want to investigate the effect of boundary (induced by the surface orientation) on the phonon transport. What is more, it is worth pointing out that, the NWs in our simulations are perfect. The mass variance (*g*) will be zero if there is no defect in our models, and then the defect scattering will have no effect on the results. Here, we consider the defect scattering in our theoretical model, so that the results predicted by the theoretical model can be more general.

Furthermore, the size effects of NWs were discussed systematically using theories based on the relaxation approximation of BTE as well. F. Alvarez *et al.*^50^ considered the size effect via doing the moment expansion of linearized Boltzmann transport equation in the relaxation time approximation, and found that the effective thermal conductivity will increase and then converge with the effective length. In the system with small effective length (dozens of nanometers in our paper), the thermal conductivity will be size dependent due to the fact that the phonons with MFP larger than the system will be truncated and have no contribution to the total thermal conductivity^35^. Recently, Hua and Cao^51^ generated a theory based on the phonon BTE to study the influence of boundary constraints on thermal conductivity of NWs. Apart from the phonon boundary scattering mechanism, they found that, the phonon ballistic transport, which usually exists in the system with length shorter or comparable to the phonon MFP, can cause temperature jump at the boundaries in contact with phonon baths. However, in our study we do not consider such effect, since the length of the models is the length that excludes the phonon baths. Both F. Alvarez *et al.*^50^ and Hua’s^51^ work show that the thermal conductivity of NWs increases firstly and converges finally with the effective length (including both the longitudinal and lateral directions), which is in accordance with our findings. Furthermore, the converged thermal conductivity of NWs can be obtained through linear extrapolation, since the MFPs of Si NWs in our simulations are only several tens of nanometers.

## Analysis of Simulation Results and Phonon Transport Mechanism

### Vibrational density of states analysis

**Figure 2:**
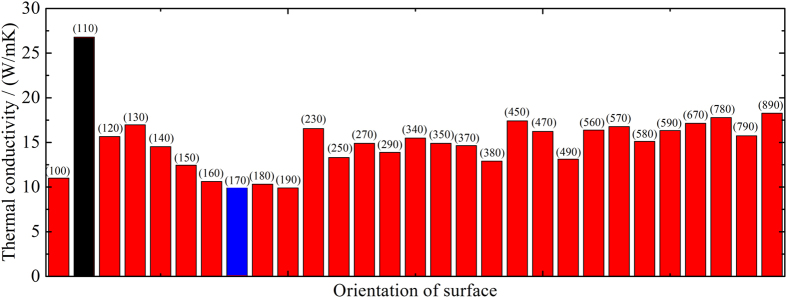
Thermal conductivity of Si nanowires with different surface orientations. The highest and lowest thermal conductivity is colored in black and blue, respectively.

**Figure 3:**
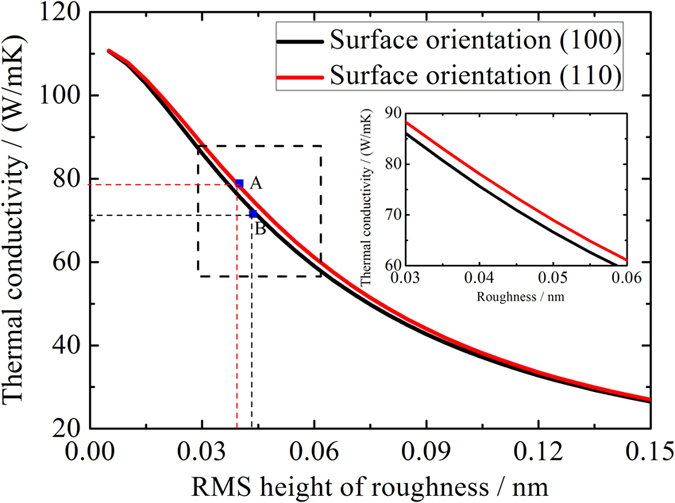
Thermal conductivity of Si nanowires as a function of RMS height of roughness for the cases of (100) and (110) surface orientation. (Inset) The dashed region is zoomed in.

**Figure 4:**
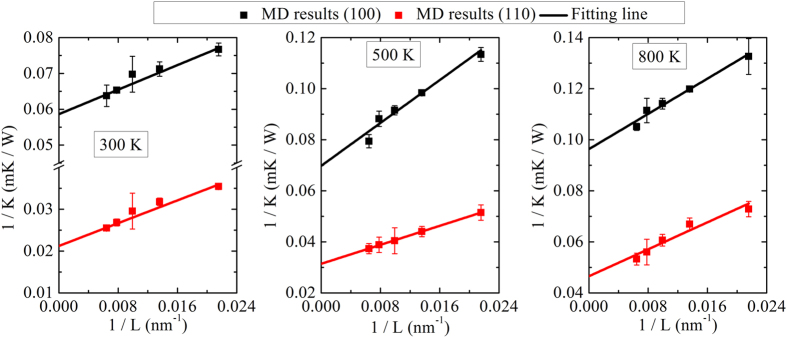
Length dependence of thermal conductivity of Si nanowires with two typical (100) and (110) surface orientations. Three different temperatures of 300 K, 500 K, and 800 K are considered.

**Figure 5:**
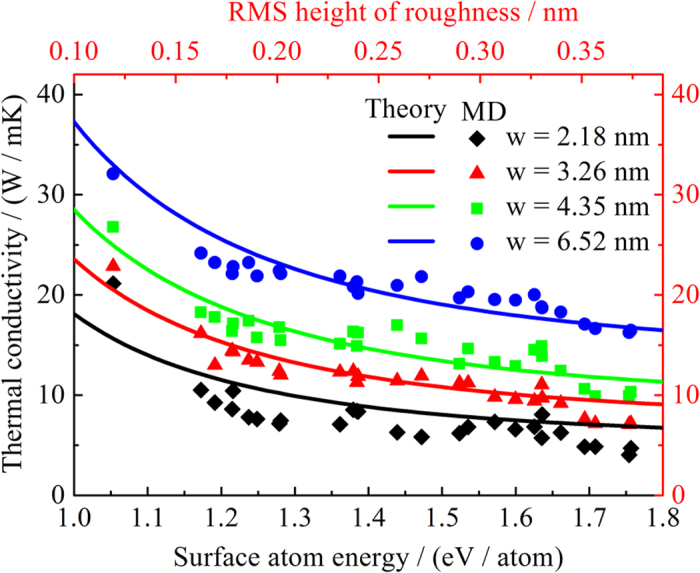
Thermal conductivity of Si nanowires with different cross-section vs. average surface atom energy. The symbols and solid lines are NEMD simulation results and theoretical predictions, respectively.

**Figure 6:**
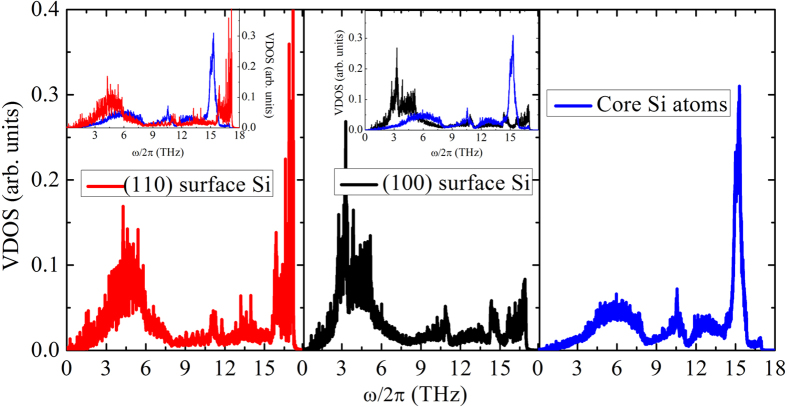
Vibrational density of states (VDOS) of surface and core atoms in Si nanowires with two typical (100) and (110) surface orientations. (Inset) Comparison of VDOS between the surface and core atoms.

To understand the mechanism of large difference between the NWs with diverse surface orientations, firstly we calculate the VDOS via Fourier transform of the autocorrelation function of atomic velocity. We compare the VDOS of Si atoms in the surface region and in the interior region (we call them core atoms in this paper) in Fig. 6. The atomic velocities of the surface and core atoms are sampled every 2 fs during the total output time of 100 ps. It can be seen that the VDOS of the atoms in the surface region have similar shapes with that in the interior region for the medium frequency range (6~14 THz), but quite different geometries in the low and high frequency range (0~6 and 15~18 THz). For the cases of both (100) and (110) surface orientation, the low frequency VDOS of the surface atoms moves leftward (redshift) and is enhanced as compared with that of the core atoms. For the core atoms, there are four typical peaks in the VDOS, which corresponds to the transverse acoustic, longitudinal acoustic, transverse optic and longitudinal optic phonon modes, respectively. Phonons of Si atoms in the (110) surface region are mainly concentrated in the low frequency region (0~7.5 THz) and a few high frequency peaks occur in the range from 9 to 18 THz. In contrast, the low frequency VDOS of Si atoms in the (100) surface region have a further left shift (redshift) as compared to that of the core atoms and the Si atoms in the (110) surface. Furthermore, the extremely high frequency VDOS (15~18 THz) for the case of (100) surface decreases largely and a new peak appears in the low frequency region (0~6 THz) with respect to in the case of (110) surface, which means the degenerated transverse acoustic phonon modes are split. Meanwhile, it can be found the overlap area of VDOS between the atoms in the (110) surface and the core atoms is larger than that between the atoms in the (100) surface and the core atoms (see insets in Fig. 6), and the overlap area mainly resides in the low frequency region (0~10 THz). In other words, the vibrational mismatch between the atoms in the (110) surface region and the core atoms is smaller with respect to other NWs considered in this paper, or equivalently, the phonon-boundary scattering for the case of (110) surface orientation is weaker, which naturally leads to the significantly higher thermal conductivity than other cases of surface orientations, as presented in Fig. 2.

### Spectral thermal conductivity

**Figure 7:**
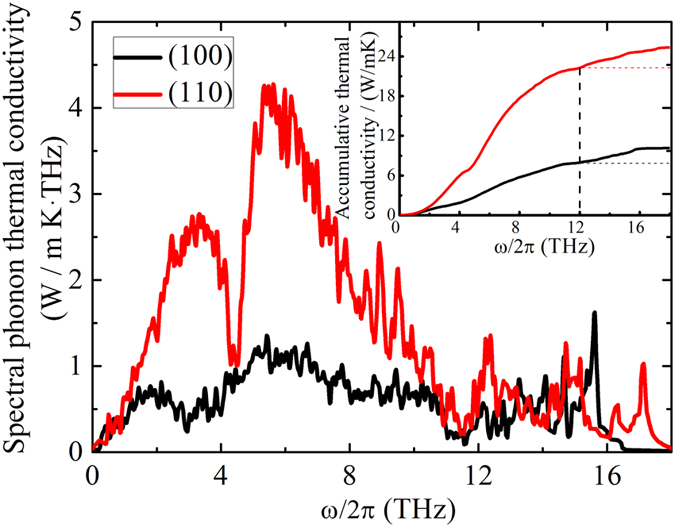
Comparison of spectral thermal conductivity of Si nanowires between (100) and (110) surface orientation at 300 K. (Inset) Accumulative thermal conductivity as a function of mode frequency. The dashed lines are guide for eyes.

Figure 7 shows the spectral (frequency dependent) thermal conductivity of Si NWs with (100) and (110) surface orientations at 300 K. It can be easily found that the difference in the frequency dependent thermal conductivity resides in the medium frequency range (1.5~10 THz), which should be responsible for the large difference in the overall thermal conductivity as shown in Fig. 2. Furthermore, the low frequency phonons (0~2 THz), or equivalently, the phonons with long MFP, contribute little to the total thermal conductivity. The medium frequency phonons (2~12 THz) contribute more than 85% of the total thermal conductivity (see inset in Fig. 7), and therefore, it proves the common sense that the phonons in the high frequency range (optic phonons) contribute little to the thermal conductivity, as discussed above. Meanwhile, we find that the contribution of phonons in the NW with (100) surface is more or less uniform in the dominant frequency range of 2 to 10 THz, in contrast to the two major peaks for NW with (110) surface, since the phonon-boundary scattering is stronger in the former case, which is caused by the rougher surface [see the structure in Fig. 1b]. We can also see that the maximum (cutoff) frequency is about 17.8 THz for NW with (110) orientation and 16.5 THz for the NW with (100) orientation, which means the NW with (110) surface is stiffer. Same conclusion has been drawn by Yang *et al.*^39^ using MD simulations.

## Summary and Conclusions

To summarize, we have calculated the thermal conductivity of Si NWs with diverse surface orientations using NEMD simulations and the full spectrum theory. The difference of the thermal conductivity among all the NWs considered can be as large as about 2.7~4.2 times. The physics responsible for this phenomenon originates from two effects: (1) different surface orientation can change the angle between the phonon wave vector and the normal vector of the surface, then alter the modal specularity and the time that a phonon needs to travel ballistically before hitting the boundary, and therefore, leads to the change of thermal conductivity; (2) surface roughness changes due to the different surface structure, and thus, the thermal conductivity varies remarkably. The latter effect is found to be the main mechanism for the large difference in the thermal conductivity. Furthermore, the thermal conductivity of Si NWs is found to decrease with surface atom energy, or RMS height of roughness. Size effects, which include both the cross-section and length, are also considered. The large difference among the NWs with diverse surface orientation is also found to exist for infinitely long NWs. We explain the underlying mechanism from phonon level using the vibrational density of states and spectral thermal conductivity. We find that, in the case of (110) orientation the mismatch of the VDOS between the surface atoms and the bulk atoms is smaller than that in other models, which means the stronger coupling between the surface and core atoms, and therefore, it leads to the highest thermal conductivity for (110) orientation among all the NWs studied. Meanwhile, with the help of our newly developed frequency domain direct decomposition method, we investigate the frequency dependent contribution of thermal conductivity. The largely scattered thermal conductivity among the NWs with various surface orientation is found to mainly originate from the medium frequency phonons (1.5~10 THz THz). The results presented herein are expected to be quite useful in thermal engineering applications, such as realizing high efficiency thermoelectrics by reducing thermal conductivity with engineered surface orientation.

## Theory and Computational Methods

### Theory of full spectrum

By using the relaxation time approximation (RTA) to solve the Boltzmann transport equation (BTE), one can obtain the relaxation time of a phonon mode (*ν*, **q**) as^52^


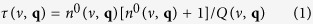


where n^0^(*ν*, **q**) is the Bose-Einstein distribution function of phonons and Q(*ν*, **q**) is the diagonal part of the three phonon-phonon, phonon-impurity and phonon-boundary operator collision, ν and **q** are the branch index and phonon wave vector, respectively. Here, one should note that the Bose-Einstein distribution can be an approximate to Boltzmann distribution, which is usually used to depict the classic system when temperature of system is moderate and high (above 300 K in our cases). The detail expression of Q(*ν*, **q**) can be written as^53^


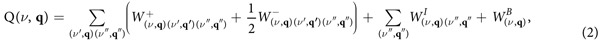


Here, 
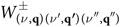
 are the scattering rates for three-phonon process. 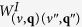 and 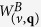 are the scattering rates for phonon-impurity and phonon-boundary, respectively. As we know, the first summation in Eq. (2) includes two processes: one is the N process which must satisfy the momentum and energy conservation simultaneously; and the other is the U process which only needs to satisfy the energy conservation. Then, the *Q* (*ν*, **q**) can be written in the form of


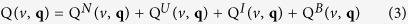


Combining with Eq. (1), we can know the total relaxation time *τ*(*ν*, **q**) reflects the Matthiessen’s rule as


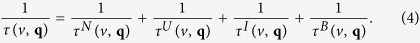


Meanwhile, the total thermal conductivity *k* can be regarded as the summation of the contribution of all phonon modes


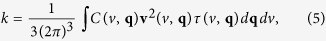


where the mode specific heat capacity is C(*ν*, **q**) = *k*_*B*_[h*ω*(*ν*, **q**)]^2^*n*^0^(*ν*, **q**)[*n*^0^(*ν*, **q**) + 1] and v(*ν*, **q**) is the group velocity of the phonon mode (*ν*, **q**).

It is well known that the relaxation time of *N* and *U* processes is dependent on both frequency and temperature: *τ*^*N or U*^(*ν*, **q**; *T*) = 1/*f*^* N or U*^[*ω*(*ν*, **q**)] ⋅ *f*^* N or U*^(*T*), where *f*^* N or U*^[*ω*(*ν*, **q**)] and *f *^*N or U*^(*T*) are the functions of frequency and temperature. Using the *ab initio* approach, Ward and Broido^54^ give the detailed expression for acoustic phonons as


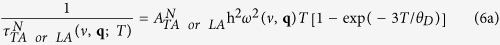



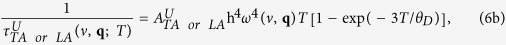


where the *θ*_*D*_ is the Debye temperature. For Si 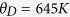, 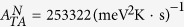, 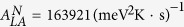, 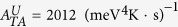 and 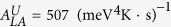.

For the contribution of optic phonons, we take the form of relaxation time of *N* and *U* processes from the Callaway model^47^


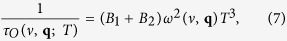


where *B*_*1*_ and *B*_*2*_ are the constants related to the three phonon-phonon scattering, *i* = 1 stands for the *N* process and *i* = 2 is the *U* process scattering. For Si Callaway obtained the parameters *B*_1_ + *B*_2_ = 3.8 × 10^−24^ (s ⋅ deg^−1^) via fitting the theoretical results to experiments^47^.

Point defect scattering from atoms with different mass is one of the most common cases of impurity scattering in materials. For such case, Klemens^55^ gives the relaxation time in the form of


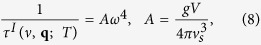


with the constant *A* containing the mass variance (*g*), the atomistic volume (*V*) and the averaged sound velocity (*v*_*s*_).

The boundary scattering is found to be related to the roughness and the orientation of the boundary^21^. The expression can be written as


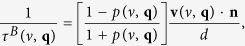


where *d* is the distance between the boundaries, *p*(*ν*, **q**) and **n** are the momentum dependent specularity parameter^39^ and direction vector of the boundary, respectively. Thus, for *p*(*ν*, **q**) = 0 which means a very rough surface, it can be regarded as the fully diffusive boundary scattering. The ideal smooth surface will have *p*(*ν*, **q**) = 1. For boundary with root mean square (RMS) height of Δ, the parameter *p*(*ν*, **q**) is calculated as^21^,^56^


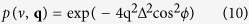


where *ϕ* is the angle between the phonon wave vector and the direction vector of the boundary surface.

Moreover, for NW with square-like cross-section, the relaxation time of phonon-boundary scattering is direction dependent, since the length of the system is usually finite. For the length of the system (*L*) and the lateral width (*W*) such terms should be in the form of


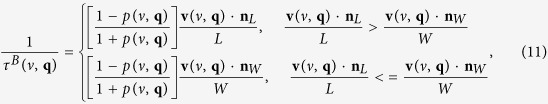


where *L*/**v**(*ν*, **q**) ⋅ **n**_*L*_ and *W*/**v**(*ν*, **q**) ⋅ **n**_*W*_ are the time that a phonon needs to travel ballistically before hitting the boundary.

### Computational details of NEMD simulations

Our large-scale nonequilibrium molecular dynamic simulations, performed with LAMMPS package^57^ and the Tersoff potential^58^, consider the Si NW systems with different surface orientation. Based on the previous experiments^26^,^27^, the Si NWs can be aligned along the [100], [110] and [111] direction with longitudinal dimension ranging from 1 to 100 nm. In our study, we focus on the [100] longitudinally oriented Si NWs with diverse surface orientations, such as (100), (110), and (890) (28 different surface orientations in total). The size of the cross-section ranges from 2.18–6.52 nm and the NW length ranges from 54.3–162.9 nm. Figure 1a shows the schematic of the simulation model. Non-periodic boundary condition is applied in all three directions. All the simulations in this paper are performed with 1.02 × 10^7^ NEMD steps with timestep of 1 fs, i.e. the total running time of MD simulation is 10.2 ns. The first 200 ps running with *NPT* (constant particles, volume, and temperature) ensemble is used to obtain the equilibrium state and the relaxed structure of Si NWs. Following the equilibrium, we run 7 ns to make sure that a steady heat flux is established in the system. To generate the heat current through the systems, the atoms located in the distance *L*_*bath*_ from the left and right ends of the system [see Fig. 1a] are coupled to hot and cold Langevin thermostats with constant temperature of *T* + Δ*T*/2 and *T* − Δ*T*/2, respectively. The left-most and right-most layers are maintained at fixed positions to prevent large deformations and the translational movement of the system. The average system temperature in all our simulations is fixed at *T* = 300 K and the temperature difference between the cold and hot thermostats is Δ = 60 K. Once the heat flux reaches steady state, we apply the frequency domain direct decomposition method (FDDDM) in the last 3 ns for the system and then obtain the heat current spectrum. The computational details of FDDDM can be found in our previous paper^59^. Finally, the thermal conductivity is obtained using the Fourier’s law. The group velocity and frequency of phonons used in our theoretical model are calculated by *ab initio*, which can provide the accurate phonon dispersion of bulk Si.

### Details of frequency domain direct decomposition method

To gain more insight into the mechanism for the effect of surface orientation on the thermal conductivity of Si NWs, we calculate the spectral thermal conductivity using the frequency domain direct decomposition method which is proposed by us recently^59^. Here, we give a brief description of FDDDM and the details can be found in ref. 59. The spectrum of heat current in a control volume can be obtained as


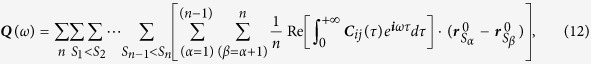


where 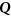 is the heat current, the sum is taken over all atoms which are described by *n*-body potential in the control volume, *s*_*i*_ denotes the atoms, 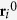 are the equilibrium position of atoms, respectively, **C**_*ij*_ is the auxiliary correlation term and can be written in the form of


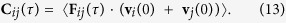


where **F**_*ij*_ is the force between two atoms *i* and *j*, **v** is the velocity of atoms, 〈 〉 denotes the time average. Then, the spectral (frequency dependent) thermal conductivity can be calculated by Fourier’s law


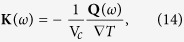


where V_*c*_ and ∇*T* are the volume of the control box [see schematic in Fig. 1a] and the temperature gradient in the NEMD simulation, respectively.

## Additional Information

**How to cite this article**: Zhou, Y. *et al.* Strong Surface Orientation Dependent Thermal Transport in Si Nanowires. *Sci. Rep.* **6**, 24903; doi: 10.1038/srep24903 (2016).
